# The application of dexamethasone implants in uveitis treatment

**DOI:** 10.3389/fmed.2024.1402396

**Published:** 2024-06-28

**Authors:** Tian Zhang, Zhutao Liu, Na Li

**Affiliations:** Affiliated Hospital of Shandong Second Medical University, School of Clinical Medicine, Shandong Second Medical University, Weifang, China

**Keywords:** uveitis, Ozurdex, intravitreal implant, macular edema, drug therapy, dexamethasone

## Abstract

Uveitis refers to a group of ocular inflammatory diseases that can significantly impair vision. Although systemic corticosteroid therapy has shown substantial efficacy in treating uveitis, extensive use of corticosteroids is associated with significant adverse effects. Recently, a biodegradable, sustained-release implant, namely dexamethasone intravitreal implant (Ozurdex), has been reported for treating non-infectious and infectious uveitis. This review aims to summarize the experiences with Ozurdex treatment across various forms of uveitis and to assist readers in understanding the appropriate timing and potential side effects of Ozurdex in uveitis treatment, thereby maximizing patient benefits in uveitis management.

## 1 Introduction

Uveitis, a group of intraocular inflammatory diseases, is categorized as anterior, intermediate, posterior, and panuveitis according to the Standardization of Uveitis Nomenclature (SUN) (1). Uveitis is further divided into infectious and non-infectious types. Uveitis can lead to complications such as macular edema, vitritis, retinal vasculitis, cataract, and glaucoma, with macular edema being the primary cause of vision loss in these patients (2). In developed countries, uveitis is the fifth most common cause of vision impairment, accounting for about 10%–15% of all blindness cases and 5%–10% of legally blind cases (3). It predominantly affects individuals in their working ages of 20–60 years, thereby significantly impacting society and healthcare systems (4).

Corticosteroids are commonly used in uveitis treatment, functioning by blocking various inflammatory cytokines, thereby inhibiting the inflammatory response, reducing capillary leakage, inflammatory cell migration, and fibrous deposition, effectively alleviating macular edema (5). Corticosteroids can be administered via various routes, including topical eye drops, periocular injections, intravitreal injections, and systemic oral therapy. The therapeutic efficacy varies depending on the drug penetration associated with each administration route (6). Water solubility is a crucial factor influencing the pharmacokinetics of corticosteroids. Dexamethasone, due to its higher water solubility, has enhanced drug loading and bioavailability but a reduced vitreous half-life, necessitating a sustained-release system to maintain long-term levels in the vitreous body (7). Ozurdex® (dexamethasone implant, Allergan Pharmaceuticals, Irvine, CA, United States), a preservative-free intravitreal implant containing 0.7 mg of dexamethasone, enables sustained drug release, ultimately degrading into water and carbon dioxide (8). Animal studies have demonstrated that Ozurdex reaches peak drug concentration in the eye around 2 months, with efficacy lasting up to 6 months (9); its pharmacokinetics are similar in vitrectomized and non-vitrectomized eyes (10, 11). These findings have been corroborated by clinical research of Pelegrín et al. (12), which indicated similar effectiveness of Ozurdex in treating non-infectious uveitic macular edema in vitrectomized and non-vitrectomized eyes; however, an increased risk of elevated intraocular pressure (IOP) was noted in the non-vitrectomized group. Ghosn et al. (13) found that intravitreal implantation of Ozurdex reduces inflammation in anterior and intermediate uveitis in animal eye models. Currently, the U.S. Food and Drug Administration (FDA) has approved Ozurdex for the treatment of macular edema due to vein occlusion, diabetic macular edema, and non-infectious uveitis. Moreover, reports suggest the potential application of Ozurdex for treating other ocular diseases, such as macular edema associated with rhegmatogenous retinal detachment, proliferative vitreoretinopathy, and post-traumatic proliferative vitreoretinopathy (14–17). This article summarizes the experiences of using Ozurdex in the treatment of various types of uveitis, discussing the optimal timing for its use and potential side effects.

## 2 Research methods

We searched the PubMed database using the keywords: “uveitis,” “Ozurdex,” “intravitreal implant,” and “dexamethasone.” Relevant articles discussing the application of Ozurdex in uveitis treatment were selected through randomized controlled trials, case reviews, and other research methods. Inclusion criteria comprised full-text articles in English and publications from this century. Articles were initially selected by screening titles and abstracts, excluding those that did not meet the review’s objectives or lacked sufficient data for a thorough evaluation. Additionally, references from related articles were collected to supplement the search. All obtained studies were categorized under the application of Ozurdex in uveitis, summarizing and discussing its role in uveitis treatment.

## 3 Clinical trials of dexamethasone implants in uveitis treatment

The HURON trial (18), the first large-scale prospective randomized controlled trial, divided patients with intermediate and posterior uveitis into three groups: 77 in the 0.7 mg dexamethasone implant group, 76 in the 0.35 mg group, and 76 in a sham surgery group. The primary endpoint was the proportion of eyes with a vitreous haze score of 0 at week eight. Results demonstrated sustained anti-inflammatory effects up to 26 weeks, with 47% in the 0.7 mg group, 36% in the 0.35 mg group, and 12% in the sham group (*P* < 0.001). The group receiving the dexamethasone implant showed a more significant improvement in best-corrected visual acuity (BCVA). However, increased rates of cataracts and elevated IOP were noted. Due to the recurrent nature of uveitis, some patients required multiple Ozurdex implants. A multicenter retrospective study by Zarranz-Ventura et al. (19) tracked the number of Ozurdex implants in 63 patients with non-infectious uveitis, totaling 82 eyes and 142 implants over 35 months. The study indicated significant improvements in vitreous haze and BCVA. However, the drug exhibited a limited duration of action, necessitating repeat implants in some cases, with 40.7% undergoing a second implant and 11.2% requiring three or more. Zola et al. (20) found that the reduction in central macular thickness (CMT) with a second Ozurdex implant was similar to the first, proving the effectiveness of repeated implants. Teja et al.’s retrospective analysis (21) showed that for uveitic macular edema unresponsive to anti-vascular endothelial growth factor (anti-VEGF) treatment, Ozurdex implantation reduced CMT and improved BCVA, suggesting its use in anti-VEGF resistant cases. A retrospective analysis by Breitbach et al. (22) noted post-Ozurdex implantation improvements in uveitic macular edema unresponsive to systemic corticosteroids. The LOUVRE 2 (23) study found that in patients with uveitis persisting for 5 years and previously treated with oral medication, Ozurdex implantation reduced CMT and improved vision. Alba-Linero et al. (24) reported that patients previously treated with systemic corticosteroids required fewer Ozurdex implants and that the use of Ozurdex can reduce systemic medication dosages.

## 4 The application of dexamethasone implant in various forms of uveitis

### 4.1 Vogt-Koyanagi-Harada

Vogt-Koyanagi-Harada (VKH) disease, an autoimmune condition targeting melanocytes (25), presents with exudative retinal detachment, optic disc edema, retinitis, and vitritis, often accompanied by neurological and cutaneous symptoms. The treatment of VKH typically requires systemic corticosteroids, which can yield significant adverse reactions. Latronico et al. (26) first reported the use of Ozurdex in treating a case of bilateral refractory VKH in a 15-year-old female. After being diagnosed with VKH, the patient received a pulse therapy of methylprednisolone 1 g/day for 5 days, followed by oral prednisone 25 mg/day with gradual tapering. Although her condition improved within a month, macular edema recurred after 2 months. Subsequently, she underwent another round of methylprednisolone pulse therapy combined with oral immunosuppressants; however, the macular edema worsened again after 1 month. Consequently, Ozurdex was implanted in both eyes concurrently with the methylprednisolone pulse therapy, leading to the resolution of macular edema without recurrence during follow-up. Chen et al. (27) reported two cases of VKH, treated post-diagnosis with corticosteroid pulse therapy combined with Ozurdex. The oral corticosteroid dosage was reduced to 5 mg/day after 2 months and discontinued after 4 months. Both patients regained a vision of 20/20 within 1 month and maintained stable conditions over 13 and 6 months of follow-up, respectively. Elhamaky (25) evaluated the efficacy and safety of Ozurdex during the chronic recurrent phase of VKH. The study included 16 patients with 29 eyes, all previously treated with corticosteroids and immunosuppressants for over 6 months. Over 24–26 months of follow-up, CMT and BCVA improved. Twenty-one eyes (72.4%) required only a single implantation, while eight eyes (27.6%) needed a second implant, with an average of 1.2 ± 0.6 implants per eye. Controlled elevation in IOP occurred in three eyes, and cataract progression was observed in 11 eyes. The results indicate that Ozurdex significantly reduces CMT and improves BCVA in the chronic recurrent phase of VKH, reducing dependence on systemic medications. Using systemic corticosteroids solely is less effective in treating macular edema and serous retinal detachment associated with VKH syndrome (26). However, combining Ozurdex has shown better therapeutic outcomes. Additionally, the literature indicates that systemic corticosteroids combined with anti-VEGF therapy yield favorable results in treating serous retinal detachment in VKH (28). Ozurdex has been proven effective as an adjuvant treatment for VKH, reducing adverse reactions associated with systemic medications and accelerating vision recovery. Although Ozurdex is effective for ocular manifestations of VKH, it is essential to consider that VKH is a systemic disease that may also affect the nervous system, hearing, skin, and hair. Therefore, careful monitoring of the patient’s overall disease course is necessary.

### 4.2 Behcet’s disease

Behcet’s disease is a multisystem inflammatory disease of unclear origin, primarily characterized by oral and genital ulcers, skin lesions, and ocular involvement (29, 30) Approximately 70% of patients with Behcet’s disease suffer from ocular complications, leading to recurrent, non-granulomatous uveitis (30). Coskun et al. (31) evaluated the efficacy of Ozurdex in the treatment of posterior uveitis associated with Behcet’s disease. The study encompassed 17 eyes from 12 patients with Behcet’s eye disease, all of whom had active posterior uveitis despite systemic corticosteroid or immunosuppressive therapy. These patients, after a single implantation of Ozurdex, discontinued corticosteroids within 1 month and continued only with immunosuppressants, with a 12-month follow-up. The results indicate an improvement in vitreous haze, a reduction in CMT, and an enhancement in BCVA. Inflammation did not recur in three eyes over the 12 months; the effect of the single injection lasted for an average of 6.9 months, reducing the need for systemic medications. Additionally, Fabiani et al. (32) assessed the efficacy of Ozurdex combined with a systemic medication in five patients with unilateral Behcet’s disease uveitis. During the six-month follow-up, retinal vasculitis resolved, CMT decreased, and BCVA improved, with only one case of uveitis recurrence. Yalcinbayir et al. (33) evaluated the effectiveness of Ozurdex in treating macular edema associated with Behcet’s disease. This study included 27 eyes from 20 patients who continued to experience macular edema despite immunosuppressive or biologic therapy. After the implantation of Ozurdex and a six-month follow-up, a reduction in CMT and an improvement in BCVA were observed. Tao et al. (34) investigated the safety and efficacy of Ozurdex as an adjuvant treatment for Behcet’s uveitis. The study included 80 eyes from 61 patients, with 50 eyes receiving Ozurdex treatment and 30 eyes serving as the control group, with a 12-month follow-up. Results show that post-Ozurdex implantation, improvements were observed in BCVA, fluorescein fundus angiography, vitreous inflammation, and CMT, with statistically significant improvements in fluorescein fundus angiography and vitreous inflammation. These findings suggest that Ozurdex can be an effective adjunctive treatment for Behcet’s disease-associated uveitis, reducing the need for systemic medications and thereby minimizing drug-induced adverse effects.

### 4.3 Sarcoidosis

Sarcoidosis, a multisystem disorder, exhibits the highest rate of ocular involvement at 79%, with uveitis being the predominant ocular manifestation. Approximately two-thirds of patients experience a chronic and recurrent course of the disease (35). Myung et al. (36) reported a case of a patient with sarcoidosis presenting with optic disc inflammation and retinal vasculitis in the left eye, who was treated with oral prednisone 60 mg and subconjunctival injection of triamcinolone. Despite this treatment, inflammation persisted in the left eye, and similar symptoms developed in the right eye. Consequently, Ozurdex implants were administered in both eyes, and oral corticosteroids were discontinued, resulting in inflammation resolution and no recurrence over the next 6 months. Zarranz-Ventura et al. (19) conducted a multicenter retrospective study on Ozurdex treatment for non-infectious uveitis, including six patients with sarcoidosis-related uveitis, showing reduced vitritis, decreased CMT, and improved vision acuity. Kim et al. (37) conducted a retrospective study with the largest sample size on Ozurdex treatment for sarcoidosis-related uveitis. The study included 20 patients, with a median follow-up of 16.5 months. Results indicate a decrease in CMT, an improvement in BCVA, and effects lasting up to 6 months. A total of 65% of the patients received one injection, while 35% required more than two injections. At the time of the first Ozurdex implant, 70% of patients were also receiving a systemic medication, which was reduced to 40% after 3 months. Sarcoidosis-associated uveitis often has a prolonged and recurrent course, making long-term systemic medication a burden physically and financially for patients. Ozurdex can serve as an adjunctive treatment for sarcoidosis-associated uveitis, potentially reducing the reliance on systemic medications.

### 4.4 Juvenile idiopathic arthritis

Juvenile idiopathic arthritis (JIA) is a common systemic disease in children, associated with uveitis and accompanied by extra-articular impairments, including ocular damage, growth disturbances, and muscle atrophy. The primary ocular manifestation in JIA is uveitis, which accounts for about 12% of cases (38, 39). Pichi et al. (40) conducted a retrospective analysis of the efficacy of Ozurdex in treating JIA-associated uveitis. The study included 16 eyes from 10 patients with JIA who had persistent uveitis despite prior treatments with corticosteroids, immunosuppressants, or biologics. The results revealed that after Ozurdex implantation, there was a reduction in anterior chamber inflammation, a decrease in CMT, and an improvement in BCVA. Twelve eyes underwent a second implantation after 7.5 ± 3.1 months, and five eyes had a third implantation after 7 ± 4.6 months from the second one. The primary adverse effects were increased IOP and progression of cataracts. Jinagal et al. (41) evaluated the safety and efficacy of Ozurdex implantation combined with cataract surgery in patients with JIA. The study included eight eyes from six patients, with a six-month follow-up period. The results indicate that all patients experienced improved BCVA and that no recurrences of uveitis were observed. The combined treatment also reduced the use of systemic corticosteroids in patients with JIA. These studies indicate that Ozurdex is effective in treating JIA-associated uveitis and can be used perioperatively to enhance the safety of intraocular surgeries. The treatment of JIA often necessitates a systemic medication; systemic corticosteroid therapy in children can lead to growth retardation, severe Cushingoid features, and associated psychosocial issues. Thus, the use of Ozurdex can reduce the risk of these complications.

### 4.5 White dot syndromes

White dot syndromes comprise a group of diseases with unclear pathogenesis, presenting significant challenges in diagnosis and treatment, typically managed with oral corticosteroids. Miserocchi et al. (42) reported the efficacy of Ozurdex in treating serpiginous choroiditis in a study of eight eyes from seven patients. All patients underwent previous treatments with corticosteroids and immunosuppressants; however, they experienced recurrences and could not tolerate increased doses of systemic corticosteroids. Results show controlled inflammation and reduced corticosteroid use, though vision improvement was not statistically significant. Corticosteroids rapidly control acute inflammation in serpiginous choroiditis, preventing scarring and recurrence, making Ozurdex a viable option for patients intolerant to systemic corticosteroids. Walsh et al. (43) reported three cases of bilateral birdshot chorioretinopathy treated with Ozurdex, showing improvements in BCVA, reduced vitreous haze, and decreased CMT, with improved quality of life due to reduced systemic medications. However, Bajwa et al. (44) later noted that while Ozurdex improved visual function and ocular inflammation in birdshot chorioretinopathy, long-term adverse effects and disease progression necessitated a switch to systemic immunosuppressive therapy. Barnes et al. (45) reported the efficacy of Ozurdex in patients with acute zonal occult outer retinopathy. In a study of six eyes with acute zonal occult outer retinopathy, treated with Ozurdex and observed for at least 1 year, intravitreal Ozurdex effectively stabilized the condition. However, because initial visual acuity was 20/30 or better, benefits were mainly observed in improved autofluorescence and reduced systemic medication. Mora-Cantallops et al. (46) reported a case of acute posterior multifocal placoid pigment epitheliopathy. Initially monitored due to good vision, the patient’s condition progressed after 2 months with vision decline. Refusing oral corticosteroid treatment, the patient received Ozurdex, resulting in ellipsoid zone recovery, vision improvement, stability in 1 month, and resolution in 3 months. The diagnosis and treatment of white dot syndromes are challenging. However, the literature suggests that Ozurdex can serve as an adjunctive therapy for white dot syndromes, effectively avoiding the adverse effects associated with systemic corticosteroids and immunosuppressants.

### 4.6 Ocular tuberculosis

Ocular tuberculosis (TB) presents with macular edema, retinal vasculitis, serpiginous choroiditis, choroidal tuberculoma, and subretinal abscess (47, 48). During anti-tuberculosis treatment, the exposure of antigens following the death of *Mycobacterium tuberculosis* can enhance the immune response, leading to an exacerbated inflammatory reaction, known as a “paradoxical reaction” (49). The concomitant use of corticosteroids or immunosuppressants during anti-tuberculosis therapy can mitigate the damage caused by delayed hypersensitivity reactions (50–52). Agarwal et al. (47) conducted a retrospective analysis of Ozurdex effectiveness in treating tubercular uveitis. The study involved 19 eyes from 17 patients with tubercular uveitis, all of whom underwent anti-tubercular treatment combined with Ozurdex implantation; the oral corticosteroid dosage was gradually reduced to discontinuation post-implantation. After 3 months, patients showed reduced vitritis, decreased CMT, and improved BCVA. Paradoxical reactions observed in two patients subsided within a month post-Ozurdex implantation. Hasanreisoglu et al. (53) reported a case where Ozurdex was used to treat a paradoxical reaction in tuberculous uveitis. The patient started anti-tuberculosis treatment and oral methylprednisolone, which was gradually tapered. Six weeks into the treatment, the patient experienced a sudden worsening of vitreous haze and macular edema leading to decreased vision. Although periocular injection of triamcinolone reduced the vitreous haze, the macular edema persisted; however, it resolved after Ozurdex implantation, with the condition stabilizing and no recurrence over a 10-month follow-up. Baharani (54) conducted a retrospective analysis on using Ozurdex alone as an adjunct in anti-tubercular treatment. The study included 13 eyes from 11 patients with tubercular uveitis, who received Ozurdex implants within 2 weeks of starting anti-tubercular treatment without oral corticosteroids. Over an average follow-up of 18.4 months, patients showed improvement in vitritis, reduced CMT, and increased BCVA. These studies demonstrate that Ozurdex is effective as an adjunct in the anti-tubercular treatment of TB ocular disease, reducing the need for systemic corticosteroids. Ozurdex can be used for paradoxical reactions during anti-tubercular treatment. Corticosteroids carry the risk of reactivating TB; localized implantation of dexamethasone implants can lower the risk of systemic TB recurrence. Before implanting Ozurdex in the vitreous cavity, concurrent anti-tuberculosis medication should be ensured; other infectious causes of uveitis should be ruled out to reduce the risk of recurrence of infectious ocular diseases.

### 4.7 Acute retinal necrosis syndrome

Acute retinal necrosis syndrome (ARNs) is a severe intraocular inflammation caused by viruses, characterized by extensive panuveitis, retinal vasculitis, and retinal necrosis (55). Late complications of ARNs include macular edema and chronic vitritis, which are often recurrent and affect vision (56). Usually, intraocular inflammation subsides within 6 to 12 weeks of initiating treatment; however, macular edema caused by ARNs can recur, posing a treatment challenge (57). Antivirals combined with corticosteroids is a common approach for managing ARNs. Previously, anti-VEGF agents were used to treat macular edema caused by ARNs; however, the results were inconsistent (57). Majumder et al. (57) reported two cases of Ozurdex being used to treat macular edema secondary to ARNs. After diagnosis, patients received a systemic antiviral combined with corticosteroid therapy. The condition was controlled; however, both patients developed macular edema in the 3rd and 4th month post-treatment, respectively. Thus, Ozurdex was implanted in both patients, along with long-term systemic antiviral therapy, resulting in the resolution of the macular edema; no recurrence of viral retinitis was observed during the follow-up period. Sørland et al. (58) reported a case of ARNs where the patient underwent a vitrectomy for a large retinal tear caused by ARNs and cataract surgery a year later. Post-surgery, the patient developed refractory macular edema. Oral prednisolone at 60 mg/day was effective in alleviating macular edema; however, a reduction to 20 mg/day led to a recurrence. Intravitreal aflibercept injections did not improve the condition. Consequently, Ozurdex was implanted in combination with oral antiviral therapy, resulting in the resolution of macular edema within 4 weeks. Over 18 months, five Ozurdex implants were administered without any recurrence of viral retinitis. A comparative study by Hu et al. (59) showed that patients with ARNs treated with a vitrectomy combined with Ozurdex had significantly lower inflammation duration, eye pressure elevation, frequency of silicone oil injection, and incidence of complications compared to the vitrectomy-only group. Ozurdex can be used as an adjunctive treatment after antiviral therapy for ARNs, proving effective for refractory macular edema. Patients with ARNs who undergo a vitrectomy combined with Ozurdex implantation demonstrate a better prognosis. Corticosteroids carry a risk of reactivating the virus; their clinical use should involve a careful assessment of the patient’s condition.

### 4.8 Ocular toxocariasis

Ocular toxocariasis (OT), a rare disease commonly seen in young patients, is associated with a history of contact with cats and dogs. It can cause macular cyst-like edema, vitritis, retinal granuloma, and retinal detachment, severely impacting the patient’s vision (60). The entry of Toxocara into the eye triggers an immune response, leading to inflammation and permanent scarring. Symptoms may include reduced vision, redness, pain, floaters, and photophobia (61). The main treatments for OT include oral corticosteroids, antiparasitic drugs, and surgery (62). Cai et al. (63) reported the first case of using Ozurdex to treat exudative retinal detachment associated with OT. A 13-year-old patient with OT developed exudative retinal detachment and retinal vasculitis. Following oral albendazole treatment combined with Ozurdex implantation, the exudative detachment resolved, without inflammation recurrence over 8 months. Although the retinal structure improved, vision only increased from counting fingers to 20/400. Zhang et al. (64) reported a case of peripheral granulomatous OT in an eight-year-old healthy boy who presented with a one-year history of vision loss, characterized by vitreous opacity, increased CMT, peripheral granuloma of the ciliary body, and mild ciliary detachment due to vitreous traction. Following a diagnosis of peripheral granulomatous OT, the patient was treated with oral albendazole 400 mg and Ozurdex implantation. Two months postoperatively, his vision improved from 20/400 to BCVA 20/100, with reduced vitreous opacity and CMT. Three months later, BCVA decreased to 20/200, and vitreous opacity worsened, prompting a second Ozurdex implantation. The patient’s vision gradually improved to BCVA 20/50, with decreased vitreous opacity and CMT. Six months after the second implantation, his vision remained stable. A study by Liu et al. (65) compared the effectiveness of oral corticosteroids vs. Ozurdex implantation in patients with OT post-vitrectomy. The results showed no significant differences in visual improvement, cataract formation, or macular pucker between the two groups. However, 86.5% of patients in the oral corticosteroid group developed obesity, while those in the Ozurdex group experienced higher IOP. Sun et al. (60) assessed the safety and efficacy of Ozurdex in treating OT-associated uveitis. The study included 78 patients with OT, with 51 receiving Ozurdex treatment and 27 serving as controls. The results indicate that Ozurdex implantation improved vitreous haze in patients with OT, enhancing BCVA in those without macular involvement; no significant adverse reactions were observed. These studies demonstrate that Ozurdex is effective in treating OT-related uveitis, reducing the physiological and psychological adverse effects associated with systemic corticosteroid use. Notably, the treatment of ocular toxoplasmosis requires intravitreal injection of clindamycin combined with dexamethasone (66). However, there are currently no reports of intravitreal dexamethasone implants in ocular toxoplasmosis, which merits future attention.

### 4.9 IRVAN syndrome

Idiopathic retinal vasculitis, aneurysms, and neuroretinitis syndrome (IRVAN syndrome) is a rare, bilateral vision-threatening disease (67). IRVAN syndrome often leads to exudative retinal detachment and extensive retinal non-perfusion areas, followed by the development of neovascularization and recurrent vitreous hemorrhages, severely impacting vision (67, 68). Empeslidis et al. (69) reported a case of IRVAN syndrome where the patient experienced persistent macular edema post-vitrectomy, following systemic corticosteroids and pan-retinal photocoagulation treatment for recurrent vitreous hemorrhages. After Ozurdex implantation, there was a reduction in CMT and an improvement in BCVA, without recurrence or adverse effects noted during a four-month follow-up. Saatci et al. (70) also described a case of IRVAN syndrome. Despite four anti-VEGF injections, the patient continued to exhibit serous retinal detachment and exudation around the optic disc. The condition did not improve after oral azathioprine and completion of pan-retinal photocoagulation treatment. However, following Ozurdex implantation, the serous retinal detachment, and peripapillary exudation diminished. The treatment of IRVAN syndrome currently lacks consensus; Ozurdex has shown effectiveness in treating macular edema and serous retinal detachment associated with IRVAN syndrome. However, due to the limited number of cases, further research is needed for the treatment of IRVAN syndrome.

### 4.10 Sympathetic ophthalmia

Sympathetic ophthalmia (SO) is an autoimmune pan-uveitis, typically induced by intraocular surgery or open-globe trauma (71). Its exact pathogenesis remains unclear. Studies suggest that this disease may be triggered by ocular trauma or surgery, which exposes normally sequestered ocular antigens to the systemic immune system, resulting in to a T-cell-mediated autoimmune response (72). The preferred treatment for SO is oral high-dose corticosteroids or immunosuppressants, typically for at least 1 year (73). Mahajan et al. (71) first reported the efficacy of intravitreal corticosteroid sustained-release implants in patients with SO. In their study, eight patients with SO received fluocinolone acetonide (Retisert) implants and were followed up for 6 months to 2 years. The results show a significant reduction in systemic medication use; five patients could completely discontinue systemic immunosuppressants. Two patients experienced recurrent inflammation requiring additional oral immunosuppressants. Three patients had improved vision, while five maintained stable visual acuity. Mansour et al. (74) reported a case of SO treated with Ozurdex. The patient, diagnosed with SO, refused oral immunosuppressants and chose Ozurdex implantation. Due to recurrent inflammation, the patient received an Ozurdex implant every 3 months, totaling six implants over an 18-month follow-up period. During this time, the patient was treated solely with Ozurdex without oral medications, improving BCVA. Although IOP increased, it was controllable with anti-glaucoma medications. These findings suggest that Ozurdex is an effective treatment for SO. While systemic corticosteroids are the first-line treatment for SO (75), long-term systemic steroid therapy can cause significant adverse effects. Using Ozurdex can reduce the need for systemic medication.

### 4.11 Radiation maculopathy

Radiation maculopathy is a condition that arises after radiation therapy for choroidal melanoma and other intraocular tumors. Common complications include macular edema, optic disc edema, and exudative retinal detachment. Typical treatments include intraocular laser, photodynamic therapy, and intravitreal injections of triamcinolone or anti-VEGF agents, with varying efficacy (76–79). Caminal et al. (80) conducted a retrospective analysis of the effectiveness of Ozurdex in treating radiation maculopathy following plaque radiotherapy for choroidal melanoma. Among the 12 patients in the study, nine patients had previously undergone laser photocoagulation or anti-VEGF treatments with suboptimal results. Over a follow-up period of 8.2 ± 7.8 months, a reduction in CMT and improvement in macular edema were observed, although the increase in BCVA was not statistically significant. Baillif et al. (81) also reported the efficacy of Ozurdex in treating radiation-induced macular edema. In their study, all five patients exhibited reduced CMT, with three experiencing visual improvement. In a case reported by Bui et al. (82), a patient who showed a poor response to anti-VEGF treatment and intravitreal triamcinolone acetonide injection experienced normalization of macular structure and stable vision following Ozurdex implantation. Stringa et al. (83) described a case where a single Ozurdex implant significantly reduced CMT and markedly improved vision over a 16-month recurrence-free follow-up. Malclès (84) conducted a retrospective analysis on the use of Ozurdex in treating exudative retinal detachment associated with choroidal melanoma. The study included 10 patients, seven of whom had exudative retinal detachment before radiotherapy and three developed it post-therapy. Over an average follow-up of 9.9 months, improvement in exudative retinal detachment was observed at 3.1 months post-implantation in seven patients, with complete resolution in six patients. At the end of the follow-up, half of the patients exhibited stable vision while the other half experienced deterioration, without adverse effects observed. Russo et al. (85) reported a case of radiation-induced macular edema. The patient did not respond to anti-VEGF treatment; however, within 4 weeks post-Ozurdex implantation, BCVA improved, and CMT decreased. These studies indicate that Ozurdex can be used in the treatment of radiation maculopathy and tumor-related exudative retinal detachment. Given the typically poor prognosis of radiation maculopathy, Ozurdex offers a promising therapeutic option for these conditions.

## 5 Complications

### 5.1 Cataract

Cataract, a common complication following the vitreous cavity implantation of Ozurdex, is frequently reported in uveitis treatment. However, the reported incidence of cataracts varies across different studies, potentially due to variations in study inclusion criteria, patient health status, and previous systemic medication use. Table 1 summarizes the progression and surgical interventions of cataracts in patients treated with Ozurdex for uveitis (Table 1).

**Table 1:** The progression and surgical situations of cataracts in patients with uveitis after using Ozurdex.

Author	The number of eyes	Follow-up period (months)	Cataract progression	Cataract surgery
Rajesh et al. (86)	2,736	18	835 (47.1%)	576 (32.5%)
Nagpal et al. (87)	116	6	9 (7.8%)	-
Lowder et al. (18)	62	6.5	9 (15%)	1 (1.6%)
Ratra et al. (88)	42	19.2	-	6 (14.3%)
Palla et al. (89)	20	12	-	5 (20%)
Tomkins-Netzer et al. (90)	38	17.3	1	-

### 5.2 Elevated IOP

IOP is another common complication following the implantation of Ozurdex. Risk factors include a history of glaucoma, higher baseline IOP, younger age, previous instances of increased IOP after implantation, uveitis, and the use of higher doses of steroids (91). There is variability in the reported cases of increased IOP following Ozurdex implantation. Table 2 summarizes the instances of increased IOP after the use of Ozurdex and their respective treatment approaches (Table 2).

**Table 2:** Rate of intraocular pressure (IOP) elevation and treatment after application of Ozurdex.

Author	The number of eyes	Follow-up period (months)	increased IOP (mmHg)	IOP lowering treatment	Glaucoma surgery
Zarranz-Ventura et al. (19)	82	35	>21 in 33 eyes (40.2%)	32 (39%)	2 (2.4%)
Zarranz-Ventura et al. (92)	159	24	>21 in 15 eyes (9.4%) >10 increase from baseline in nine eyes (5.7%)	19.6%	-
Malclès et al. (93)	421	16.8	>25 or an increase of ≥10 from baseline (28.5%)	12 (31%)	3 (0.7%)
Ratra et al. (88)	42	19.2	>21 in seven eyes (16.6%)	7 (16.6%)	1 (2.4%)
Nobre-Cardoso et al. (94)	58	6	>21 in 21 eyes (36.2%)	21 (36.2%)	0

### 5.3 Endophthalmitis

Intravitreal implantation of Ozurdex carries a risk of intraocular infection. The incidence of infectious endophthalmitis following Ozurdex implantation varies across different studies. A retrospective analysis by Samuelson et al. (95), which included 3,925 Ozurdex implantations, reported four cases of endophthalmitis, yielding an incidence rate of 0.102%. Another study by Stem et al. (96) reviewed 3,593 Ozurdex implantations in 1,051 patients, identifying five cases of endophthalmitis, with an incidence rate of 0.14% (per injection) and 0.4% (per patient). Pancholy et al. (97) compared the risk of endophthalmitis following Ozurdex implantation with the risk after injections of 0.5 mg and 0.3 mg of ranibizumab. Out of 4,973 Ozurdex implantations, there were five cases of endophthalmitis (0.1%), whereas 43 cases of endophthalmitis occurred out of 163,974 injections of 0.5 mg ranibizumab (0.026%), and six cases out of 18,954 injections of 0.3 mg ranibizumab (0.031%).

### 5.4 Abnormal implant location

With the widespread clinical use of Ozurdex, cases of abnormal implant positioning have been increasingly reported. Accidental migration of the implant to the subconjunctival space is a rare but serious complication, potentially resulting in corneal edema and decompensation (98). Fenolland et al. (98) reported an instance where the injector was inadvertently inserted into the subconjunctival space, breaking the implant into three parts that remained under the bulbar conjunctiva. The dexamethasone implant was not immediately removed during the procedure, resulting in corneal endothelial decompensation after 3 weeks; the patient’s BCVA deteriorated from 20/60 to 20/200. The cornea regained transparency, and BCVA improved to 20/60 after surgical removal of the implant. Ong et al. (99) also reported a case of Ozurdex migration into the subconjunctival space following injection. In this patient, prompt removal of the implant prevented complications. Additionally, intravitreal implants can migrate into the anterior chamber. Betsch et al. (100) reviewed 32 cases of implants entering the anterior chamber, all in patients with artificial intraocular lenses. Of these, 21 cases (65.6%) underwent removal of the implant, six cases (18.8%) had the implant repositioned back into the vitreous cavity, and 12 (37.5%) eventually required corneal transplantation. Risk factors for migration into the anterior chamber include vitrectomy, capsular bag rupture, aphakia, and iridectomy. Implant migration into the lens is a rare complication that can potentially accelerate cataract formation. Biswas et al. (101) reported such a case, where the patient ultimately underwent combined cataract surgery and vitrectomy. In case of improper implantation technique, or if the ocular fluid resistance decreases, the implant may enter the vitreous cavity too quickly, causing iatrogenic retinal damage. Kim et al. (102) reported a case where the implant penetrated the retina and choroid during insertion; however, no complications were observed during a 12-month follow-up.

## 6 Conclusion

In this study, we have thoroughly summarized and discussed the application of dexamethasone implants in uveitis treatment, along with a statistical analysis of related complications. Ozurdex has shown efficacy in various types of uveitis, providing precise anti-inflammatory effects that help reduce adverse reactions associated with systemic corticosteroids. It exhibits significant efficacy, particularly in treating vitreous inflammation and macular edema caused by uveitis. Additionally, Ozurdex usage can improve patient compliance and reduce economic burden, offering a new option for patients with systemic diseases, pregnant patients, and those intolerant to systemic drug side effects. However, potential complications such as cataracts, glaucoma, and implant displacement require careful attention during treatment; infection risks should be cautiously excluded before implantation.

## Author contributions

TZ: Conceptualization, Writing – original draft, Writing – review & editing. ZL: Writing – review & editing. NL: Conceptualization, Methodology, Supervision, Writing – review & editing.
